# Approaching Thrombospondin-1 as a Potential Target for Mesenchymal Stromal Cells to Support Liver Regeneration after Partial Hepatectomy in Mouse and Humans

**DOI:** 10.3390/cells13060529

**Published:** 2024-03-17

**Authors:** Lysann Tietze, Madlen Christ, Jiyeon Yu, Peggy Stock, Sandra Nickel, Annelie Schulze, Michael Bartels, Hans-Michael Tautenhahn, Bruno Christ

**Affiliations:** 1 Department of Visceral, Transplant, Thoracic and Vascular Surgery, University of Leipzig Medical Center, 04103 Leipzig, Germany; lysann.tietze@medizin.uni-leipzig.de (L.T.); madlen.christ@medizin.uni-leipzig.de (M.C.); peggy.stock@medizin.uni-leipzig.de (P.S.); sandra.nickel@medizin.uni-leipzig.de (S.N.);; 2 Klinik für Allgemein-, Viszeral- und Thoraxchirurgie, Helios Park-Klinikum Leipzig, 04289 Leipzig, Germany; jiyeon.yu@helios-gesundheit.de (J.Y.); michael.bartels@helios-gesundheit.de (M.B.); 3 Division of General, Visceral and Vascular Surgery, Jena University Hospital, 07747 Jena, Germany; 4 Research Programme “Else Kröner-Forschungskolleg AntiAge”, Jena University Hospital, 07747 Jena, Germany

**Keywords:** partial hepatectomy, post-hepatectomy liver failure, liver regeneration, human mesenchymal stromal cells, THBS1, cell transplantation

## Abstract

Extended liver resection carries the risk of post-surgery liver failure involving thrombospondin-1-mediated aggravation of hepatic epithelial plasticity and function. Mesenchymal stromal cells (MSCs), by interfering with thrombospondin-1 (THBS1), counteract hepatic dysfunction, though the mechanisms involved remain unknown. Herein, two-thirds partial hepatectomy in mice increased hepatic THBS1, downstream transforming growth factor-β3, and perturbation of liver tissue homeostasis. All these events were ameliorated by hepatic transfusion of human bone marrow-derived MSCs. Treatment attenuated platelet and macrophage recruitment to the liver, both major sources of THBS1. By mitigating THBS1, MSCs muted surgery-induced tissue deterioration and dysfunction, and thus supported post-hepatectomy regeneration. After liver surgery, patients displayed increased tissue THBS1, which is associated with functional impairment and may indicate a higher risk of post-surgery complications. Since liver dysfunction involving THBS1 improves with MSC treatment in various animal models, it seems feasible to also modulate THBS1 in humans to impede post-surgery acute liver failure.

## 1. Introduction

In humans, liver resections of up to 70–80% of liver mass are demanded in cases of primary liver tumors or liver metastases. This is critical, because the quality of the remaining hepatocytes is decisive for future liver remnant function in terms of proliferative and metabolic performance. These, in turn, may be impaired due to the patient’s individual attributes like sex, age, co-morbidities, or neoadjuvant tumor therapy [1,2].

Platelets support liver regeneration after partial liver resection in mice and humans [3]. Fibrinogen-driven platelet aggregation in the liver promoted liver restoration, while, vice versa, abrogation of fibrinogen deposition and/or platelet aggregation was associated with increased post-operative liver failure and mortality [4]. Accordingly, post-surgery low platelet abundance correlates with a delay in liver regeneration after partial resection [5], poor prognosis of recovery, and increased incidence of post-operative liver failure [6,7]. Platelets might beneficially impact on liver regeneration by stimulation of proliferation of endothelial cells secreting pro-regenerative factors like hepatocyte growth factor (HGF) and IL6 upon interaction with platelets [8,9].

There is an obvious inconsistency between the role of platelets and platelet-derived factors affecting liver regeneration after partial hepatectomy (PHx). The promotion of liver regeneration by platelets as described above seems contradictory to the retardation of regeneration by platelet α-granule-derived cytokines. Platelet-derived thrombospondin-1 (THBS1) was reported to be a negative predictor of post-hepatectomy liver function in humans [10,11]. This was also shown in THBS1-knockout mice, in which liver regeneration improved after PHx by stimulation of hepatocyte proliferation and mitigation of hepatocyte apoptosis as compared to wildtype animals [12]. This suggests a suppressor role of THBS1, likely mediated by THBS1 activation of latent TGF-β, presumably a negative modulator of liver regeneration after PHx [12]. These findings are in line with the role of TGF-β as an enhancer of epithelial plasticity of the hepatic parenchyma during liver regeneration [13,14].

Recently, mesenchymal stromal cells (MSCs) were shown to support liver regeneration after extended partial hepatectomy in rodent [15,16,17,18] and pig [19,20] animal models. Mechanistically, MSCs attenuated platelet recruitment to the liver and downstream THBS1-mediated TGF-β activation and epithelial disruption. Yet, the primary mechanism behind this process remained open [21]. Here, we want to examine the hypothesis of whether MSCs influence liver surgery-induced platelet activation and their recruitment to the injured liver. Thereby, MSCs might mute platelet-mediated hepatic epithelial disruption and support liver regeneration and recovery of function.

The data shown here in a mouse model of two-thirds hepatectomy suggest that MSCs decrease platelets and macrophages in the liver. On the other hand, MSCs increase activated platelets. Thus, though MSCs attenuate platelet recruitment to the liver and THBS1 release thereof, their activation seems to foster epithelial integrity and function. Surgery-induced activation of THBS1 also seems to be involved in post-operative liver dysfunction in patients undergoing the ALPPS (Associating Liver Partition and Portal Vein Ligation for Staged Hepatectomy) procedure.

## 2. Materials and Methods

### 2.1. Animal Trials

Experiments were approved and authorized by the federal state of Saxony (file no. TVV23-20) and followed the guidelines of the Animal Welfare Act. Male C57BL/6J mice at the age of 10–14 weeks were obtained from the animal facilities of the Faculty of Medicine at the University of Leipzig (MEZ Leipzig). Housing conditions comprised a 12 h light/dark rhythm, 25 °C room temperature, and free access to water and a standard rodent chow containing 9 kJ% fat, 24 kJ% protein, and 67 kJ% carbohydrates as the energy sources (V1534, ssniff, Soest, Germany). Animals were assigned randomly to three different groups. Two groups underwent a two-thirds partial hepatectomy as described previously [22], one group receiving 1 × 10^6^ hepatocytic differentiated human bone marrow-derived mesenchymal stromal cells (hBM-MSCs) after the liver resection, and the other group receiving PBS as a vehicle control via splenic delivery essentially as described in [23]. For the calculation of cell numbers, please refer to ref. [24]. The third group of animals received laparotomy and closure of the wound only (sham-treated group). Liver samples for downstream analyses were collected at 6, 24, and 48 h post-surgery. At each point in time, the sham groups comprised 3 animals and the control and hBM-MSC-treated groups comprised 7–12 animals. The isolation and hepatocytic differentiation of MSCs from human bone marrow has been described in detail elsewhere [22] and was approved by the Institutional Ethics Review Board of Leipzig (file no. 282/11-lk).

### 2.2. Human Liver and Blood Samples from Patients after ALPPS

Permission to collect human tissue specimens was approved by the Institutional Review Boards of Leipzig and Jena (331-15-24082015 and 2018-1246). Patients were diagnosed with liver cancer and underwent the ALPPS procedure (Associating Liver Partition and Portal Vein Ligation for Staged Hepatectomy). In a first step, this surgical technique comprises portal ligation and dissection of the tumor-bearing liver lobe, leading to its atrophy, while the non-ligated parts of the liver undergo hypertrophy, thus increasing the future liver remnant volume, anticipating the augmentation of future function. Next, 1–2 weeks after the first step, the atrophic liver lobes are resected. This allows liver tissue samples to be acquired before (step 1) and at the time point of resection (step 2; resectate and remnant liver). Serum albumin levels and the activated Partial Thromboplastin Time (aPTT) were determined in the serum of patients in need of liver resection by the Institute of Clinical Chemistry and Laboratory Diagnostics, Centralised Diagnostic Laboratory Services, Jena University Hospital (UKJ).

### 2.3. Analyses of Mouse Plasma and Liver Tissue Samples

Mouse blood samples were collected by puncture (25 gauge cannula) of the vena cava inferior 6, 24, and 48 h after surgery, transferred to reaction tubes, and immediately centrifuged in a benchtop centrifuge for 5 min at 2500× *g* and 4 °C.

Aspartate aminotransferase and alanine aminotransferase activities (µkat/L) were determined at the Institute of Laboratory Medicine, Clinical Chemistry and Molecular Diagnostics, University of Leipzig Medical Center.

To determine blood concentrations of THBS1 and TGF-β3, blood samples were collected after euthanasia of the animals at the indicated time points after surgery. Samples were immediately centrifuged for 15 min at 1000× *g* to separate plasma from cells. Plasma samples were stored at −80 °C until use. Liver tissue samples (100 mg) were homogenized, stored overnight at −20 °C after two freeze–thaw cycles, and centrifuged for 5 min at 5000× *g*. Resulting supernatants were stored at −80 °C until use. THBS1 and TGF-β3 were determined in duplicate by using 100 µL of each plasma and liver supernatant according to the suppliers´ manuals. THBS1 in mouse plasma and liver tissue was measured by applying the mouse thrombospondin 1 ELISA kit (CSB-E08765m, Cusabio, Houston, TX, USA) and the mouse TSP-1 ELISA kit (EM1434, FineTest, Fine Biotech Co., Ltd., Wuhan, China), resp. Levels of TGF-β3 in plasma and tissue samples were determined by using the mouse transforming growth factor β3 ELISA kit (CSB-E12862m, Cusabio, Houston, TX, USA). Chromogen formation was measured using the GloMax^®^-Multi Detection System (Promega, Mannheim, Germany). Concentrations of TGF-β3 and THBS1 were calculated by running standards alongside the samples as exemplified in the manufacturers’ manuals.

Liver triglycerides were determined by using the Triglyceride Assay Kit-Quantification (ab65336, Abcam, Berlin, Germany) essentially as described previously [25].

### 2.4. Quantification of Ki67-Positive Nuclei

To quantify the proliferation of liver cells, paraffin-embedded liver tissue slices (1.5 µm) were stained with an anti-Ki67 antibody (1:200, ab66155, abcam, Cambridge, UK). After dewaxing, epitopes were retrieved by heating in citrate buffer (10 mM; pH 6.0) for 45 min, and endogenous peroxidases were blocked using 3% hydrogen peroxide for 20 min. Slices were then incubated in blocking solution (5% BSA and 0.5% Tween 20 in PBS) for 90 min, followed by a 20 min avidin–biotin blocking step (Vector Laboratories, Burlingame, CA, USA) and incubation with the anti-Ki67 antibody overnight at 4 °C. After three washing steps, slices were incubated for 60 min at room temperature with the secondary biotin-SP-conjugated anti-rabbit IgG antibody (1:200, 111-065-003, Dianova, Hamburg, Germany). After another washing step, slices were treated with the ABC-kit (PK-6100; Vector Laboraties, Burlingame, CA, USA) according to the manufacturer’s manual, followed by incubation with the DAB chromogen (Thermo Fisher Scientific, Dreieich, Germany) and counterstaining with nuclear fast red detection solution (Carl Roth GmbH, Karlsruhe, Germany) for 1 min. Slices were embedded in Entellan (Merck GmbH, Darmstadt, Germany) and images acquired using the Zeiss Axio Oberserver.Z1 microscope (Carl Zeiss AG, Oberkochen, Germany). To estimate proliferation, the percentage of Ki67-positive liver cells was determined by counting the number of positively stained nuclei divided by the total number of nuclei using the ImageJ 1.48v software (National Institute of Health, Bethesda, MD, USA). Three animals in the sham and five animals in the control and the hBM-MSC-treated group each were included into the analysis and 15 microscopical fields (orig. magnification—20×) were counted on one tissue section from each animal.

### 2.5. sqRT-PCR Analyses

To estimate the expression of E-cadherin and ZO-1, total RNA was isolated for sqRT-PCR. Liver tissue was homogenized in Qiazol reagent (Qiagen, Hilden, Germany) followed by chloroform extraction and isopropanol precipitation. The quality of the RNA was checked spectrophotometrically at 260 and 280 nm. RNA was further processed if the ratio at 260/280 was in the range of 1.75–1.95. Next, 4 µg of total RNA was used to generate cDNA using the Maxima H Minus First cDNA syn Kit (Thermo Fisher, Dreieich, Germany). RT-PCR was run in the PCR Mastermix 2× (Thermo Fisher, Dreieich, Germany) with the corresponding primer pairs (Supplementary Table S1). For normalization, beta-2-microglobulin (B2M) was used as a housekeeping gene. PCR products were separated electrophoretically in agarose gels and stained with GelRed^®^ (Biotium, Fremont, CA, USA). Band intensity was quantified using the ImageJ 1.48v software (National Institute of Health, Bethesda, MD, USA).

### 2.6. Immunofluorescent Co-Detection of THBS1 with CD42b, of THBS1 with CD11b, of E-cadherin (E-cad) and Zonula Occludens-1 (ZO-1), and of N-cadherin and Glutamine Synthetase (GS) in Mouse Liver

For immunofluorescence co-staining, dewaxed paraffin slices were incubated in TRIS/EDTA buffer (10 mM TRIS, 1 mM EDTA, pH 9.0) in a pressure cooker for 30 min, washed in PBS for 5 min, then blocked for 25 min in blocking solution (5% goat serum in PBS), and for another 80 min in blocking solution (5% BSA and 0.5% Tween 20 in PBS). Slices were incubated with the anti-THBS1 and the anti-CD42b or the anti-CD11b antibodies (Supplementary Table S2) in a humid chamber overnight at 4 °C and thereafter washed three times for 5 min each. The first corresponding secondary antibody labelled with Cy3 (Supplementary Table S2) was added for 50 min at room temperature and thereafter washed off two times for 10 min each. The secondary antibody labelled with Alexa Fluor 488 (Supplementary Table S2) was added for another 50 min at room temperature, followed by 3 additional washing steps for 5 min each in PBS. Nuclei were stained with DAPI (Thermo Fisher Scientific, Dreieich, Germany) and slices were mounted with 50% glycerol solution and lacquer. Images were taken with the Zeiss Axio Observer.Z1 microscope equipped with the ApoTome.2 (Carl Zeiss AG, Oberkochen, Germany).

The co-staining of E-cad with ZO-1 and N-cad with GS was carried out exactly according to the protocol, except for the use of respective primary antibodies as detailed in Supplementary Table S2.

### 2.7. Immunofluorescent Co-Detection of Cytochrome P450 Subtype 2E1 (CYP2E1) and Glutamine Synthetase (GS) in Human Liver

The co-staining of CYP2E1 and GS was carried out exactly according to the protocol as described under Section 2.6., except for the use of respective primary antibodies as detailed in Supplementary Table S2.

### 2.8. Immunohistochemical Detection of THBS1 in Human Liver

Tissue slices were incubated for 35 min in a pressure cooker in citrate buffer (100 mM citric acid, 100 mM sodium citrate, pH 6.0). Then, after cooling, they were treated with 3% hydrogen peroxide, blocked in BSA (5%), and finally in Avidin/Biotin (Vector-Kit SP-2001, BIOZOL GmbH, Eching, Germany) blocking solution. The anti-THBS1 antibody was added overnight at 4 °C (Supplementary Table S2) followed by three washing steps with PBS. The biotin-labeled secondary antibody (Supplementary Table S2) was added and slices incubated for 60 min at room temperature, followed by another three washing steps with PBS. The ABC reagent (Vector- Kit PK-6100, Vectorlabs, Burlingame, CA, USA) was applied for 30 min, slices washed in PBS, and DAB solution (Pierce™ DAB Substrate Kit, Thermo Fischer Scientific GmbH, Dreieich, Germany) added for color development. Finally, slices were counterstained with nuclear fast red (Roth GmbH, Karlsruhe, Germany), and embedded in Entellan (Merck GmbH, Darmstadt, Germany).

### 2.9. Flow Cytometry of Platelets in Mouse Liver

To estimate the percentage of activated platelets in the liver, flow cytometry was applied. The platelet fraction was detected by the CD41 marker (integrin alpha-IIb) (Supplementary Table S3). Activated platelets were determined by flow cytometric analysis of CD62P (P-selectin) (Supplementary Table S3), which was exposed to the cell membrane upon platelet activation. Thus, when imaging the total platelet fraction by CD41 expression, additional CD62P expression allows for estimation of the percentage of activated platelets out of the total [26].

Livers were passed stepwise through 400 µm, 100 µm, and 70 µm cell strainers by using the pestle of a 5 mL syringe, in a total of 10 mL of ACD buffer (sodium citrate 22.0 g/L, dextrose 24.5 g/L, citric acid 8.0 g/L, all Thermo Fisher Scientific, Dreieich, Germany). The cell suspensions were centrifuged at 100× *g* for 5 min at room temperature, and 50 µL of the supernatants were transferred to test tubes containing 50 µL of staining buffer (2% FCSi in PBS).

Liver homogenates were stained at room temperature for 45 min in the dark. The samples were then washed once in 1 mL staining buffer and centrifuged at 400× *g* for 5 min at room temperature. Pellets were resuspended in 300 µL staining buffer and analyzed. Staining conditions comprised unlabeled platelets, platelets labeled with the isotype control antibodies, and platelets stained with the anti-CD41/-CD62P antibodies, and flow cytometric analysis was performed using gates as described in [27]. For each animal, samples were run in triplicate.

Beforehand, the gate for platelet analysis was identified by Hoechst 33342 (Sigma-Aldrich, Darmstadt, Germany) exclusion combined with the size comparison using the Micron Bead Calibration Kit (1.0 µm, 3.0 µm, 6.0 µm; Bangs Laboratories, Inc., Fishers, IN, USA). In liver cell suspensions, 10,000 platelet events per sample were monitored. Analyses were executed at the Core Unit Fluorescence-Technologies, University of Leipzig, Faculty of Medicine, using the BD LSR Fortessa cell analyzer equipped with a red laser (640 nm); violet laser (405 nm); blue laser (488 nm); yellow-green laser (561 nm); and UV laser (355 nm), and the BD FACS Diva Software 9.0.1 (BD Biosciences, Heidelberg, Germany). A schematic in Supplementary Figure S1 details the gating strategy.

### 2.10. Statistics

Statistical analyses were carried out by using IBM^®^ SPSS^®^ Statistics (Version 27.0.1.0; IBM Deutschland GmbH, Ehningen, Germany). Details are outlined in the legends of figures.

## 3. Results

### 3.1. MSCs Support Post-Surgery Restoration of Tissue Homeostasis in the Mouse Liver

The De Ritis quotient denominates the ratio between serum aspartate and alanine aminotransferases (AST, ALT). A ratio >1 indicates severe hepatocellular damage, since AST and ALT are predominantly located in the mitochondria and the cytoplasm, respectively. Partial hepatectomy causes liver damage, as evidenced by the increase in the quotient at 6, 24, and 48 h post-surgery to 1.6 ± 0.34, 2.9 ± 0.33, and 3.4 ± 0.68, respectively. This increase is significantly lower in animals treated with hBM-MSCs; i.e., 1.4 ± 0.21, 1.8 ± 0.26, and 1.9 ± 0.47 at 6, 24, and 48 h, respectively, indicating less liver damage in animals treated with hBM-MSCs (for details, cf. Supplementary Table S4).

In control animals, parenchymal damage is also obvious in HE-stained liver tissue sections 48 h after partial hepatectomy. The parenchyma features widened sinusoids, necrotic hepatocytes, and tissue disintegration due to lipid accumulation. These are less obvious in the animals treated with hBM-MSCs and are comparable with sham-treated animals (Supplementary Figure S2).

**Figure 1:**
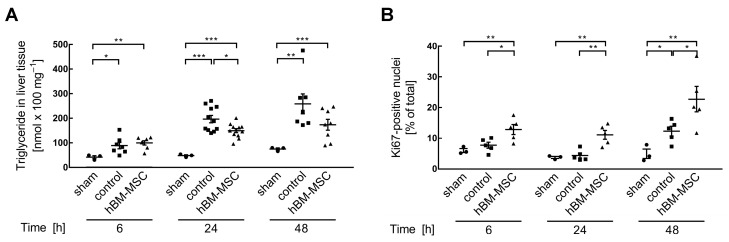
Treatment of mice with hBM-MSCs attenuates post-hepatectomy lipid accumulation and increases proliferation. (**A**) Triglycerides in liver tissue were measured by using the Triglyceride Assay Kit (Abcam); values are means ± SEM from 3 (sham) and 7–12 animals in the control and hBM-MSC groups, respectively, as indicated by the single data points. To assess for data normality, the Shapiro–Wilk and the Kolmogorov–Smirnov tests were applied. The tests revealed a deviation from normal distribution and a Johnson transformation was performed. Further statistical analysis between the different treatments was performed using Student‘s *t*-test. Values are significantly different at the *p*-levels of * ≤ 0.05, ** ≤ 0.01, *** ≤ 0.001. (**B**) Proliferating cells were identified by Ki67 staining and positive nuclei counted by using the ImageJ software as described under Material and Methods. Values are means ± SEM from 3 animals in the sham and 5 animals in the control and the hBM-MSC-treated groups each. To assess for data normality, the Shapiro–Wilk and the Kolmogorov–Smirnov tests were applied. The tests revealed a non-normal distribution and a Johnson transformation was performed. Further statistical analysis between the different treatments was evaluated using Student‘s *t*-test. Values are significantly different at the *p*-levels of * ≤ 0.05, ** ≤ 0.01. Representative images are presented in Supplementary Figure S3.

Partial hepatectomy promotes triglyceride deposition in the liver parenchyma. This provides fatty acids as an energy substrate during post-hepatectomy regeneration, but may eventually cause lipid overload due to functional impairment of the residual parenchyma [28,29]. At any point in time investigated, two-thirds hepatectomy significantly increases triglyceride accumulation as compared to the sham-treated animals. At 24 h after resection, hBM-MSCs significantly attenuate this increase, and 48 h after resection, triglyceride levels in hBM-MSC-treated animals are still lower as compared to control animals, yet not significantly (Figure 1A). This may indicate superior functional capacity in the MSC-treated as compared to untreated control animals.

In order to determine whether MSC treatment might also support liver regeneration, we determined the proliferation of liver cells by immunohistochemical detection of the nuclear proliferation marker Ki67. At 48 h after PHx, proliferation increases by 2.5- and 4.6-fold over sham-operated animals in the control and hBM-MSC-treated groups, respectively. Stromal cell treatment stimulates proliferation at 6 h after surgery, while in the control group the increase is visible only after 48 h (Figure 1B). This indicates that hBM-MSCs accelerate regeneration after PHx. This is in line with previous publications showing the augmentation of proliferation by adipose tissue-derived MSCs, which was paralleled by the increase in hepatotropic factors like hepatocyte growth factor (HGF), vascular endothelial growth factor (VEGF), and others after ischemia–reperfusion injury and partial hepatectomy in mini pigs [30].

### 3.2. MSCs Decrease Liver Tissue TGF-β3 and THBS1

**Figure 2:**
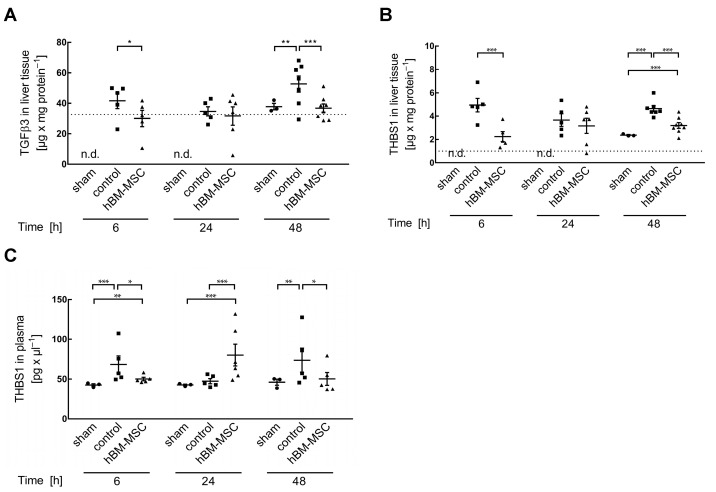
Treatment of mice with hBM-MSCs attenuates post-hepatectomy elevation of TGF-β3 and THBS1; (**A**) TGF-β3 and (**B**) THBS1 were determined in liver tissue by ELISA at the times indicated after two-thirds partial hepatectomy in sham, control, and hBM-MSC-treated animals. Values are means ± SEM from 3 animals in the sham and 5–7 animals each in the control and the hBM-MSC treatment groups. To assess for data normality, the Shapiro–Wilk and the Kolmogorov–Smirnov tests were used. Further statistical analysis between the different treatments was performed using Student‘s *t*-test. (**C**) Blood samples were taken at the time points indicated and plasma THBS1 determined by ELISA in the supernatant after centrifugation. Values are means ± SEM from 3 (sham) and 5–6 animals in the control and hBM-MSC groups, respectively, as indicated by the single data points. To assess for data normality, the Shapiro–Wilk and the Kolmogorov–Smirnov tests were used. The tests did not confirm normal distribution and a Johnson transformation was performed. Further statistical analysis between the different treatments was performed using Student‘s *t*-test. Values are significantly different at the *p*-levels of * ≤ 0.05, ** ≤ 0.01, *** ≤ 0.001. n.d.—not determined. The dotted lines represent values in liver tissue (resectates) at the time of surgery.

In the pig, MSC treatment ameliorates tissue deterioration after partial hepatectomy by inhibition of the surgery-induced increase in TGF-β1 [21], the major mediator of epithelial plasticity in the regenerating liver [14,31]. As measured in mice before surgical intervention, hepatic TGF-β3 levels amount to 32.63 ± 2.85 µg/mg protein (mean ± SEM; n = 10). This value is significantly elevated at 6 h (*p* ≤ 0.05) and at 48 h (*p* ≤ 0.001) after two-thirds hepatectomy, while no increase is obvious at 24 h. The elevation of TGF-β3 at 6 and 48 h is significantly lower after treatment with hBM-MSCs (Figure 2A). These data suggest a surgery-induced biphasic increase in hepatic TGF-β3, which is attenuated by MSCs.

THBS1 stimulates TGF-β by interacting with the latency-associated peptide, releasing the cytokine in its active form [32]. Pre-surgery levels of THBS1 are 1.01 ± 0.07 µg/mg protein (mean ± SEM; n = 10). They are significantly increased by two-thirds partial hepatectomy at 6 h (*p* ≤ 0.001), 24 h (*p* ≤ 0.001), and 48 h (*p* ≤ 0.001) after surgery, which is attenuated by the treatment with hBM-MSCs (Figure 2B). Thus, the intermittent increases of THBS1 and TGF-β are concordant with a functional relationship.

Platelets are a major source of THBS1. Activation is followed by secretion from α-granules and recruitment and aggregation at the site of injury [33,34]. Therefore, we assumed that the increase in THBS1 in the liver might be paralleled by an increase in the blood. Determination by ELISA of THBS1 in the plasma shows surgery-induced increases at 6 and 48 h post-surgery, which are attenuated by treatment with hBM-MSCs (Figure 2C). We therefore anticipate that the increase in THBS1 in the liver (cf. Figure 2B) is the consequence of the secretion of THBS1 from activated platelets either in the blood or in the liver as the consequence of a THBS1 “overflow”. TGF-β3 plasma levels remained unchanged or were even increased at 48 h upon treatment (Supplementary Figure S4). Thus, inhibition of platelet activation by hBM-MSCs in the blood or in the liver is the primary event, followed by downstream secondary effects on TGF-β in the liver.

**Figure 3:**
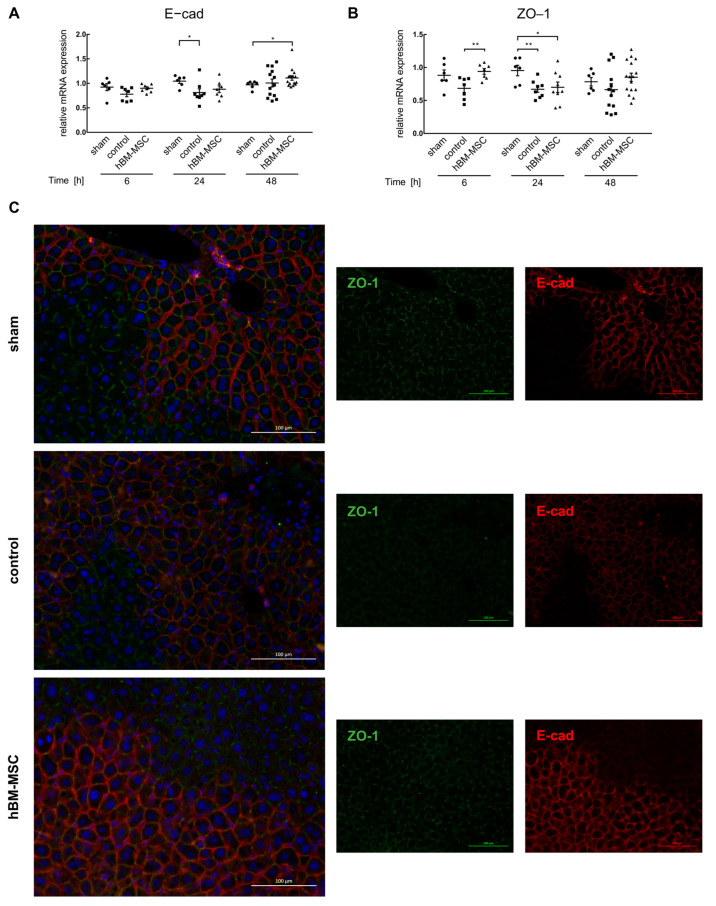
Treatment of mice with hBM-MSCs ameliorates surgery-induced attenuation of epithelial integrity. The expression of the epithelial markers E-cadherin (**A**) and ZO-1 (**B**) was estimated in liver tissue by sqRT-PCR at the times indicated after two-thirds partial hepatectomy in sham, control, and hBM-MSC-treated animals. Values are means ± SEM from 6 animals in the sham and 7–16 animals each in the control and the hBM-MSC treatment groups. To assess for data normality, the Shapiro–Wilk and the Kolmogorov–Smirnov tests were applied. Further statistical analysis between the different treatments was performed using Student‘s *t*-test. Values are significantly different at the *p*-levels of * ≤ 0.05, ** ≤ 0.01. In (**C**), E-cadherin (red fluorescence) and ZO-1 (green fluorescence) were visualized in liver tissue sections 48 h after partial hepatectomy by immunofluorescent microscopy. Left panels—overlay; middle panels—ZO-1; right panels—E-cadherin. Microscope—Zeiss Axio Observer.Z1. Original magnification—20×. Scale bar—100 µm.

In order to show whether changes in the availability of active TGF-β modulate the plasticity of the hepatic parenchyma, the epithelial markers E-cadherin and ZO-1 were determined by sqRT-PCR. Expression of E-cadherin and ZO-1 is largely slightly lower in the control group vs. the sham-operated animals, albeit mostly featuring no significant differences. Treatment with hBM-MSCs prevents that slight down-regulation, yet largely not significantly (Figure 3A,B). However, even if slight, the decrease in cell–cell contact mRNA expression suggests that epithelial integrity might be challenged by the partial hepatectomy, which could be prevented by MSC treatment, at least in part. We visualized the expression of the periportal marker E-cadherin and the zonula occludens protein component ZO-1 lining the bile canaliculi by fluorescent immunohistochemistry. In sham-treated mice, E-cadherin is expressed in periportal hepatocytes with a clear localization at the cell membrane. Likewise, ZO-1 is detected at the hepatocyte membrane pan-parenchymally as expected. In control livers treated by partial hepatectomy, the clear membraneous localization becomes blurry, featuring, in addition to the membrane, cytoplasmic localization, while periportal E-cadherin expression remains preserved. This is indicative of parenchymal relaxation after partial hepatectomy. Animals treated with hBM-MSC feature sharp zonal and membranous expression of E-cadherin and ZO-1 comparable to sham-treated animals (Figure 3C). E-cad is predominantly expressed in periportal hepatocytes and N-cadherin (N-cad) to higher levels in pericentral hepatocytes. Expression of glutamine synthetase (GS) marks hepatocytes adjacent to the central vein. Partial hepatectomy induces the loss of N-cad expression, while GS expression becomes blurry. Treatment with hBM-MSCs preserves N-cad and GS expression, indicating protection from a loss of epithelial integrity and functional quality (Supplementary Figure S5). Thus, partial liver resection causes distortion of epithelial integrity both periportally and pericentrally. Even if two-thirds hepatectomy causes only slight changes in the gene expression of epithelial markers on the RNA level, this markedly affects epithelial proteins involved in cell–cell contacts, eventually impeding epithelial integrity and potentially triggering functional impairment of the hepatocytes.

### 3.3. MSCs Promote Activation of Platelets in the Liver

**Figure 4:**
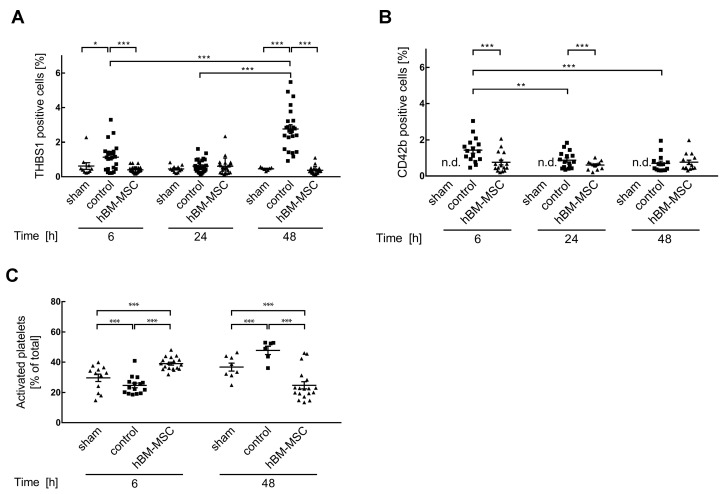
Treatment of mice with hBM-MSCs inhibits post-hepatectomy recruitment of platelets to the liver remnant. THBS1 (**A**) and platelets (**B**) were detected by double immunofluorescence using anti-THBS1 and anti-CD42b antibodies, respectively. After image acquisition (cf. Supplementary Figure S6 for representative pictures), signals were quantified using ImageJ as described in the Section 2. Values in (**A**) are means ± SEM from 2–3 animals in the sham, 4–6 in the control, and 4–5 in the hBM-MSC groups, respectively. In (**B**), 4–5 animals were included in the different groups. To assess for data normality, the Shapiro–Wilk test and the Kolmogorov–Smirnov tests were used. The tests did not confirm normal distribution and a Johnson transformation was performed. Further statistical analysis between the different treatments was performed using Student‘s *t*-test. Values are significantly different at the *p*-levels of * ≤ 0.05, ** ≤ 0.01, *** ≤ 0.001. n.d.—not determined. (**C**) The proportion of activated platelets out of the total was determined 6 h and 48 h after PHx by flow cytometry as described in the Section 2. Values are means ± SEM from 4 animals in the sham, 4–5 in the control, and 6 in the hBM-MSC groups, respectively. Post hoc analysis was conducted using the chi-square test (χ^2^) with Bonferroni correction. Values are significantly different at the *p*-level of *** ≤ 0.001.

After two-thirds partial hepatectomy, THBS1 is elevated in the liver at 6, 24, and 48 h (cf. Figure 2B). In order to confirm whether this coincides with the occurrence of platelets in the liver, THBS1 and platelets were co-detected by double immunofluorescence using an anti-THBS1 antibody and an anti-CD42b antibody as a platelet marker. Microscopically, the THBS1 increase at 6 h coincides with the accumulation of platelets. Thereafter, platelets gradually decrease until 48 h. At any point in time, hBM-MSC treatment diminishes both THBS1 and CD42b (Supplementary Figure S6). The elevated THBS1 levels at 48 h are not related to an increase in platelets, but rather to the accumulation of as yet unidentified cells, which is also attenuated by the treatment of mice with hBM-MSCs (Supplementary Figure S6). Quantitative image analysis using positive signal cell counting with ImageJ confirms increased THBS1 at 6 and 48 h and the inhibition by MSC treatment (Figure 4A). The THBS1 increase at 6 h coincides with elevated CD42b and the inhibition by hBM-MSC treatment. Platelet count thereafter significantly decreases at 24 and 48 h. While hBM-MSCs further decrease the platelet signal at 24 h, levels at 48 h are not different in control and hBM-MSC-treated livers (Figure 4B), corroborating that the increase in THBS1 at 48 h is not linked to platelets.

The immunohistochemical detection of THBS1 alone does not allow the activation state of platelets to be inferred. Therefore, we applied flow cytometry analysis using double fluorescent staining of CD41 as a platelet marker and CD62p as a marker of platelet activation to determine the percentage proportion of activated out of total platelets. At 6 h after partial hepatectomy, activated platelets amount to 39% ± 1 of total platelets in the hBM-MSC group, while in sham and control animals, the proportion is significantly lower (30% ± 2.4 and 25% ± 1.5, respectively). At 48 h, activated platelets in the sham and control groups are 37% ± 2.6 and 48% ± 2.7, respectively, while in the hBM-MSC group, activated platelets have the significantly lower proportion of about 25% ± 2.4 (Figure 4C). Taking these data together, it may be concluded that partial hepatectomy recruits platelets to the liver, which is attenuated by hBM-MSC treatment. In addition, MSC treatment supports the activation of platelets, thus accelerating tissue healing and regeneration (cf. Figure 1B). Hence, MSCs attenuate the platelet-mediated THBS1 accumulation in the liver early after surgery. Later, hBM-MSC treatment inhibits the increase in THBS1, which is mainly due to other as yet unidentified cells.

As stated before, the increased THBS1 at 48 h may not be attributed to platelets. Based on the microscopical aspect, we assumed in our first approximation that the THBS1-positive cells at this time point might represent macrophages. Double immunofluorescent staining using THBS1 and the myoloid marker CD11b identifies cells positive for both markers at 48, not at 6 h after partial hepatectomy. Thus, elevated THBS1 levels at 48 h are attributed to the accumulation of macrophages, which is attenuated by hBM-MSC treatment (Supplementary Figure S7). Since CD11b demarcates pro-inflammatory polarization of macrophages [35], we anticipate that hBM-MSC treatment attenuates the recruitment of inflammatory macrophages to the liver at later points in time after partial hepatectomy.

### 3.4. THBS1 Might Mediate Hepatic Complications after Liver Surgery in Humans

The mouse data presented here confirm our previous findings in the pig that complex liver surgery, e.g., extended liver resection, might enhance epithelial plasticity via THBS1 along the THBS1/TGF-β signaling pathway [21]. Physiologically, this is needed to allow for tissue remodeling and repair after resection, but may lead to epithelial disorganization followed by hepatocyte functional impairment under sustained elevated THBS1 [13,14].

In the pig, MSC treatment prevents a post-operative increase in THBS1 and TGF-β1, thus mitigating post-surgery complications and supporting liver regeneration [21], in line with the data shown here in the mouse. Therefore, since MSCs improve liver regeneration in pig and mouse, we anticipated that MSCs might also be a treatment option for THBS1-mediated post-operative complications in humans. To approach this hypothesis, we chose patients undergoing the ALPPS procedure, allowing for tissue acquisition at the time before the start of liver surgery (step 1) and immediately before resection (step 2, future remnant liver). Two selected patient data sets are shown here in the main manuscript. Patient no. 2 recovered from the surgical intervention and was discharged from the hospital, while patient no. 4 eventually died from liver failure among other complications. Data from another two patients with a favorable outcome may be found in the Supplementary Figures S8 and S9.

**Figure 5:**
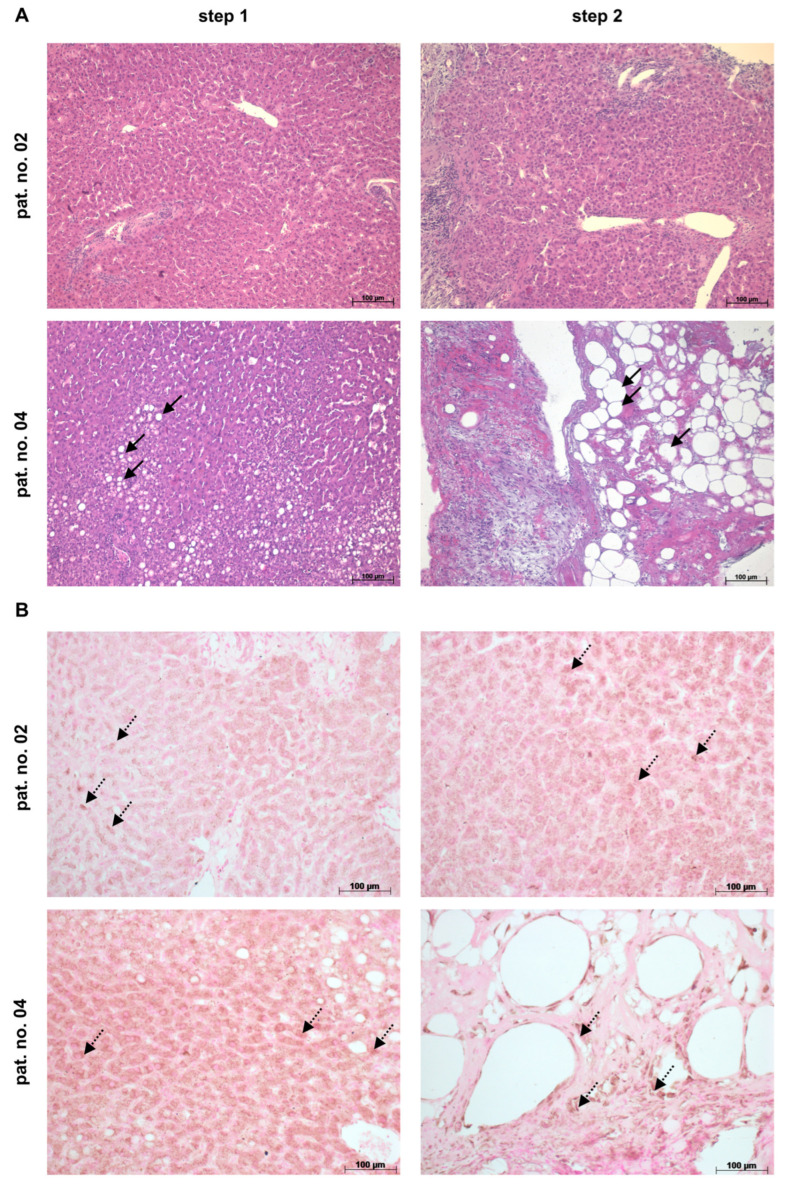
Tissue morphology (**A**) and abundance of THBS1 (**B**) in livers of patients undergoing the ALPPS procedure. Tissue samples were taken at the time points of step 1, representing the situation at the starting point, and at step 2, featuring the time point of resection of the ligated liver lobe(s). At step 2, biopsies were acquired from the future liver remnant. In patient no. 02, the HE stain in (**A**) reveals mainly regular tissue morphology at both time points, whereas the liver of patient no. 04 displays fat depositions (black arrows) at step 1, which further increase until the time point of step 2. Immunohistochemical staining of THBS1 (**B**) is already visible (brown stain, dotted arrows) at step 1 and further increases in the future liver remnant at step 2. Microscope—Zeiss Axio Observer.Z1. Original magnification—10× (**A**), 20× (**B**). Scale bar—100 µm.

The HE staining reveals patient-specific differences in tissue morphology. While patient no. 02 features nearly regular parenchymal organization at step 1, the liver tissue of patient no. 04 displays moderate fat depositions, indicating a pre-surgical fatty liver history (Figure 5A, left panels). At the time point of step 2, the future liver remnant of patient no. 02 still features quite normal tissue morphology, while in patient no. 04, the liver exhibits marked fat depositions and pathological tissue remodeling, predicting functional impairment of the future liver remnant (Figure 5A, right panels).

Since in the mouse, the increase in triglyceride content after partial hepatectomy coincides with an increase in THBS1 in the liver, we analyzed the abundance of THBS1 in the human liver tissue samples immunohistochemically in order to delineate a potential relationship. At the time point of step 1, THBS1 is detected in both liver specimens with apparently higher abundance in patient no. 04 in comparison to patient no. 02 (Figure 5B, left panels). As compared to step 1, the future liver remnant at the time point of step 2 displays a higher abundance of THBS1 in both patients. Yet, in patient no. 04, THBS1 is more pronounced and mainly detected in small cells in the pathologically destroyed areas, as well as in the direct vicinity of large lipid droplets (Figure 5B, right panels). The obvious increase in THBS1 in the future liver remnant at step 2 as seen here is corroborated by results from another two patients, as shown in Supplementary Figure S8.

### 3.5. High THBS1 Coincides with Functional Impairment of the Liver

**Figure 6:**
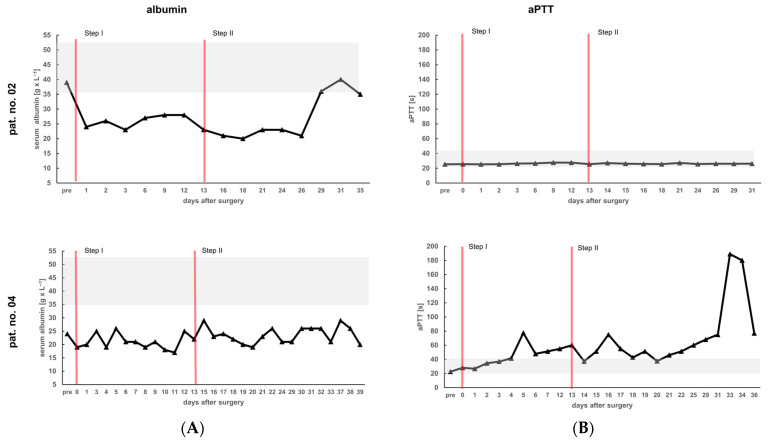
Peri-operative time course of blood levels of albumin and the activated Partial Thromboplastin Time (aPTT). At the indicated points in time of the ALPPS procedure, albumin (**A**) and aPTT (**B**) were determined in the patients´ blood samples. The single steps of the procedure are marked by red vertical lines. Grey background indicates the physiological range of values.

We hypothesized that tissue deterioration as seen in patient no. 04 might be associated with functional interference, e.g., with plasma protein synthesis. Taking serum albumin as a surrogate, post-surgery blood levels in patient no. 02 recover after surgery, while levels in patient no. 04 are consistently lower over time as compared to the other three patients (Figure 6A, Supplementary Figure S9). Also, the activated Partial Thromboplastin Time (aPTT), indicating the capacity of blood clotting, is obviously higher in patient no. 04 as compared to the other patients (Figure 6B, Supplementary Figure S9). This may indicate the functional impairment of hepatic plasma protein synthesis, including clotting factors, in this patient.

**Figure 7:**
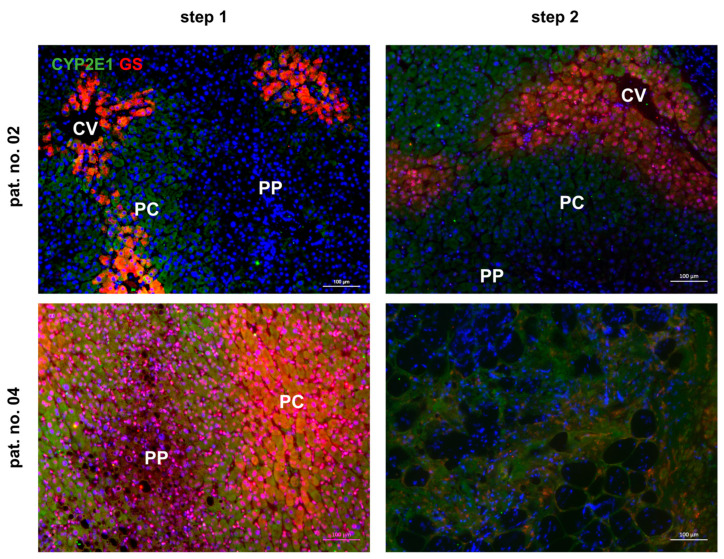
Zonal expression of CYP2E1 (green fluorescence) and GS (red fluorescence) in livers of patients undergoing the ALPPS procedure. Tissue samples were taken at the time point of step 1, representing the situation at the starting point, and at step 2, featuring the time point of resection of the ligated liver lobe(s). At step 2, biopsies were acquired from the future liver remnant. Preoperative (step 1) expression of CYP2E1 and GS in patient no. 02 features physiological pericentral zonation of the proteins, while the broad and strong expression in patient no. 04 indicates pathological enlargement of the pericentral areas. Liver histotopology in patient no. 02 is largely preserved at step 2 with pericentral expression of CYP2E1 and GS. Because of ample tissue deterioration, no clear assignment of zonal features was possible in the liver of patient no. 04. Microscope—Zeiss Axio Observer.Z1. Original magnification—10×. Scale bar—100 µm. CV—central vein. PC—pericentral hepatocytes. PP—periportal hepatocytes.

Liver function is tightly linked to the epithelial organization of the hepatic parenchyma. Hepatocytes lined along the hepatic sinusoids feature different metabolic functions, a phenomenon which is called “metabolic zonation“. E.g., in the normal liver, hepatocytes surrounding the distal branches of the portal vein execute, among other functions, gluconeogenesis and ammonia detoxification via the urea cycle, while hepatocytes adjacent to the proximal branches of the central vein predominantly perform glycolysis and ammonia fixation via the glutamine synthetase reaction [36,37]. In order to show whether functional impairment might correlate with the loss of parenchymal organization, we investigated the localization of cytochrome P450 subtype 2E1 (CYP2E1) and of glutamine synthetase (GS) by fluorescent immunohistochemistry. In patient no. 02, preoperative (step 1) expression of CYP2E1 and GS is confined to pericentral hepatocytes and hepatocytes adjacent to the central vein, respectively, as is the normal situation in the healthy liver. The zonal expression is retained at step 2, albeit demarcation is more elusive and zonal extension seems broadened. Patient no. 04 features very broad zonal expression of both CYP2E1 and GS preoperatively. At step 2, GS expression is not visible and CYP2E1 expression occurs all over the parenchyma without obvious zonal predominance (Figure 7). Thus, functional impairment seemingly correlates with epithelial distortion, which coincides with high pre- and post-operative THBS1.

Taking these findings together, we see the same situation in humans as in mice and pigs, i.e., the coincidence of high THBS1, disturbance of tissue homeostasis, and functional impairment of the hepatic parenchyma. This might indicate a causal relationship, which could be targeted by MSC treatment in order to support tissue and functional homeostasis after liver surgery.

## 4. Discussion

### 4.1. MSCs Support Mouse Liver Regeneration after Partial Hepatectomy by Mitigating THBS1

Treatment with hBM-MSCs accelerates liver regeneration after two-thirds partial hepatectomy in the mice featuring enhanced proliferation at 6 h after surgery. In contrast, a significant increase in proliferation in controls is seen only after 48 h. As compared to untreated mice, less liver damage and superior functional performance is observed in the MSC-treated animals. Thus, we anticipate that MSCs both ameliorate surgery-induced liver injury and support post-operative tissue restoration. Similar findings were reported in rats [17,38,39,40], pigs [21,41], and mice [18,42,43], indicating the versatility of MSCs in the treatment of post-hepatectomy liver failure [16].

In THBS1-knockout mice, liver regeneration after partial hepatectomy was enhanced, suggesting THBS1 is a negative regulator of regeneration [12]. There is an obvious causal relationship between THBS1 and the impact of MSCs, since the treatment of pigs with MSCs attenuated THBS1 and downstream actions on TGF-β and epithelial plasticity of the hepatic parenchyma [21]. The action of MSCs on THBS1 must occur in the liver, because post-surgery liver injury is reduced in treated animals, suggesting a protective mechanism of MSCs. We assume that MSC-mediated attenuation of tissue damage may result in less recruitment of platelets to the liver. Since the proportion of activated platelets was higher in MSC treatment at 6 h after partial hepatectomy as compared to non-treated animals, MSCs seemingly also promote the activation of platelets, which is in line with the early onset of repair of tissue damage. Mechanistically, we suggest that MSCs timely attenuate liver damage and platelet recruitment, but simultaneously stimulate platelet activation, thus supporting wound healing and liver regeneration. Vice versa, we may conclude that the sustained activation of platelets and the secretion of THBS1 and downstream TGF-β are at least part of the mechanisms fostering epithelial plasticity and post-surgery hepatic dysfunction.

Besides its role as an attenuator of liver regeneration [12], THBS1 is seemingly involved in the pathogenesis of various liver diseases. In patients suffering from acute-on-chronic liver failure (ACLF), pro-inflammatory monocytes are a major source of THBS1 involved in disease progression [44]. THBS1 impacts on liver metabolism towards lipid storage, thus supporting the pathogenesis of MASH (Metabolic Dysfunction-Associated SteatoHepatitis) in rodents [45,46] and humans [47,48,49]. In particular, pro-inflammatory monocytes/macrophages turn out to provide THBS1 as a driver of inflammatory diseases, e.g., in aneurysm tissue [50] or pulmonary hypertension due to hypoxia [51] in mice. Consistently, we identified macrophages displaying THBS1 positivity at 48 h after partial hepatectomy (cf. Figure S7) in line with macrophage-derived THBS1 as a driver of inflammatory diseases. The inhibition by MSC treatment of both platelet and macrophage recruitment to the liver after partial hepatectomy mirrors the well-documented immune regulation-related actions of MSCs on a variety of immune cells, including, among others, T cells, dendritic cells, and macrophages in general [52], and particularly in the liver [53]. Thus, we conclude that MSCs act in a protective and anti-inflammatory manner by attenuating THBS1, which eventually accelerates hepatic restoration after partial hepatectomy.

### 4.2. THBS1 Might Be a Therapeutic Target of MSCs in Humans after Complex Liver Resections

THBS1 was identified as a negative predictor of post-hepatectomy liver regeneration both in knock-out mouse models [12] and in clinical studies [11,54]. Our data corroborate these observations. However, if platelets are the main source of THBS1, its negative impact on liver regeneration contradicts clinical studies showing that post-operative low platelet count is associated with a poorer outcome [3,5]. Likewise, transfusion of platelets fostered liver regeneration after partial hepatectomy in rats [55] and humans [56]. The support of regeneration by platelets seems to rely on their interaction with liver endothelial cells. Upon interaction, platelets become activated and release growth factors like HGF and Il6, thus promoting hepatocyte proliferation directly or stimulating growth factor release from non-parenchymal liver cells [9,57].

Previous clinical studies demonstrate that platelets may promote regeneration after liver resection [58], but may also negatively affect survival after resection [54]. The inconsistency between platelet-derived pro- and anti-regenerative signals impacting on post-hepatectomy liver restoration implies that the regulation of liver regeneration after partial hepatectomy is manifold. Most plasma proteins of the coagulation cascade, also involved in platelet recruitment and activation, are synthesized in the liver [59]. E.g., due to its restricted synthetic capacity, anti-thrombin III, a potential inhibitor of the platelet activator thrombin, is diminished after partial hepatectomy, thus potentially increasing the risk of post-hepatectomy liver failure [60,61].

Besides this indirect molecular interaction regulating platelet activation or inactivation, direct cellular communications are involved comprising immune cells and particularly liver sinusoidal endothelial cells. In the pathogenesis of systemic inflammation, platelets release an abundance of stored mediators, a central phenomenon in sepsis referred to as Disseminated Intravascular Coagulation (DIC) [62]. Platelets form aggregates with leukocytes, especially at sites of endothelial inflammation, which causes a disturbance of microcirculation and increased platelet consumption in the blood, resulting in thrombocytopenia [63]. Likewise, it has also been shown in various models of acute liver failure that activated platelets reduce sinusoidal blood flow through the secretion of vasoactive mediators and promote the development of inflammatory intrahepatic microthrombosis [64].

From a clinical point of view, the disruption of microcirculation, particularly in the sinusoids by the activation of platelets and subsequent microthrombosis, must therefore be avoided to maintain adequate liver perfusion and thus function after liver resection. MSCs apparently mitigate the recruitment of platelets into the liver parenchyma (cf. Figure 4) while simultaneously accelerating their activation. Thus, the balance of platelet recruitment and state of activation rather than their absolute amount is decisive for liver function after surgery. Fine tuning between molecular and cellular communication in the liver is therefore critical for the regulation of platelet migration and activation and ultimately decisive for the aggravation of post-hepatectomy liver dysfunction or support of functional liver mass restoration. These communications still remain elusive and warrant further investigation to solve the obvious discrepancy between the beneficial and unfavorable actions of platelets in post-hepatectomy liver restoration [65].

## 5. Conclusions

The mouse model used in the present study features a major limitation. Two-thirds partial hepatectomy is not a critical situation in mice, which accept this extended resection in general without large complications and restore the loss of tissue mass in about 8–20 days [66]. Thus, this model may not reach boundary conditions for the development of post-hepatectomy liver complications. Therefore, if THBS1 is critical for survival, changes in THBS1 and downstream effects may occur to a moderate extent only. This is reflected by the fact that some of the effects measured were not significantly different between sham-treated animals and controls, and between controls and hBM-MSC-treated animals, respectively. Also, we may not clearly explain the hBM-MSC-induced increase in plasma THBS1 at 24 h post-surgery (cf. Figure 2C). This phenomenon was not observed in the liver (cf. Figure 4A). We may speculate that the MSC-induced plasma increase in THBS1 might be due to other circulating cells like immune cells, which upon stimulation by tissue injury secrete THBS1 to facilitate tissue remodeling at the site of injury [67]. A more precise resolution of the cellular sources of THBS1 and their time courses in order to define their impact on short-term healing or pathophysiological processes, respectively, under long-term conditions of sustained elevation of THBS1 is warranted.

In the present study, the changes in hepatic tissue integrity and function were comparable to effects observed in our previous study in the pig [21] and in studies presented by other groups [12]. We applied a xenogeneic cell transfusion model showing that human BM-MSCs act across species in mice, which hence may serve as a versatile animal model to allow for the further investigation of molecular pathways involving THBS1 as an attenuator of liver regeneration and identification of pathways targeted by BM-MSCs. This comprises in-depth identification of THBS1 cell sources, a more precise time resolution of THBS1-sensitive cell recruitment to the liver, and identification of downstream THBS1 molecular targets. This opens perspectives for the development of causative therapy approaches by modulating THBS1 and downstream events. However, clinical proof is mandatory, in addition to addressing patient-specific features like age, sex, co-morbidities, and pre-existing liver diseases among others.

## Data Availability

Data are contained within the article or Supplementary Material. Primary source data are available on request from the corresponding authors.

## Supplementary Materials

The following supporting information can be downloaded at: https://www.mdpi.com/article/10.3390/cells13060529/s1, Figure S1: Gating strategy for the detection of activated platelets by flow cytometry; Figure S2: hBM-MSCs attenuate deterioration of hepatic tissue integrity after two-thirds partial hepatectomy; Figure S3: Immunohistochemical detection of proliferating liver cells after two-thirds partial hepatectomy by Ki67 staining; Figure S4: hBM-MSC treatment does not change blood levels of TGF-β3 after two-thirds hepatectomy; Figure S5: hBM-MSCs attenuate deterioration of tissue integrity after two-thirds partial hepatectomy; Figure S6: hBM-MSCs attenuate thrombospondin-1-positive platelets in the liver after two-thirds partial hepatectomy; Figure S7: hBM-MSCs attenuate the increase in macrophages in the liver after two-thirds partial hepatectomy; Figure S8: Tissue morphology and abundance of THBS1 in livers of patients undergoing the ALPPS procedure; Figure S9: Post-operative time course of blood levels of albumin and the activated Partial Thromboplastin Time (aPTT) in patients undergoing the ALPPS procedure; Table S1: Summary of primer pairs and conditions used for semi-quantitative RT-PCR; Table S2: Summary of antibodies and their dilutions used in this study; Table S3: Summary of antibodies used for flow cytometry; Table S4: Summary of serum transaminases after two-thirds partial hepatectomy.
