# Supplementary material for: Approaching Thrombospondin-1 as a Potential Target for Mesenchymal Stromal Cells to Support Liver Regeneration after Partial Hepatectomy in Mouse and Humans
Source: Cells. 2024 Mar 17;13(6):529. doi: 10.3390/cells13060529 (PMC10969617; doi:10.3390/cells13060529)
Supplement: Supplementary file 1 [file cells-13-00529-s001.zip › cells-2890233-supplementary.pdf]

Approaching thrombospondin-1 as a potential target for mesenchymal stromal cells to support liver regeneration after partial hepatectomy in mouse and humans

Lysann Tietze, Madlen Christ, Jiyeon Yu, Peggy Stock, Sandra Nickel, Annelie Schulze, Michael Bartels, Hans-Michael Tautenhahn, Bruno Christ

Supplementary File

Supplementary Results

Figure S1. Gating strategy for the detection of activated platelets by flow cytometry

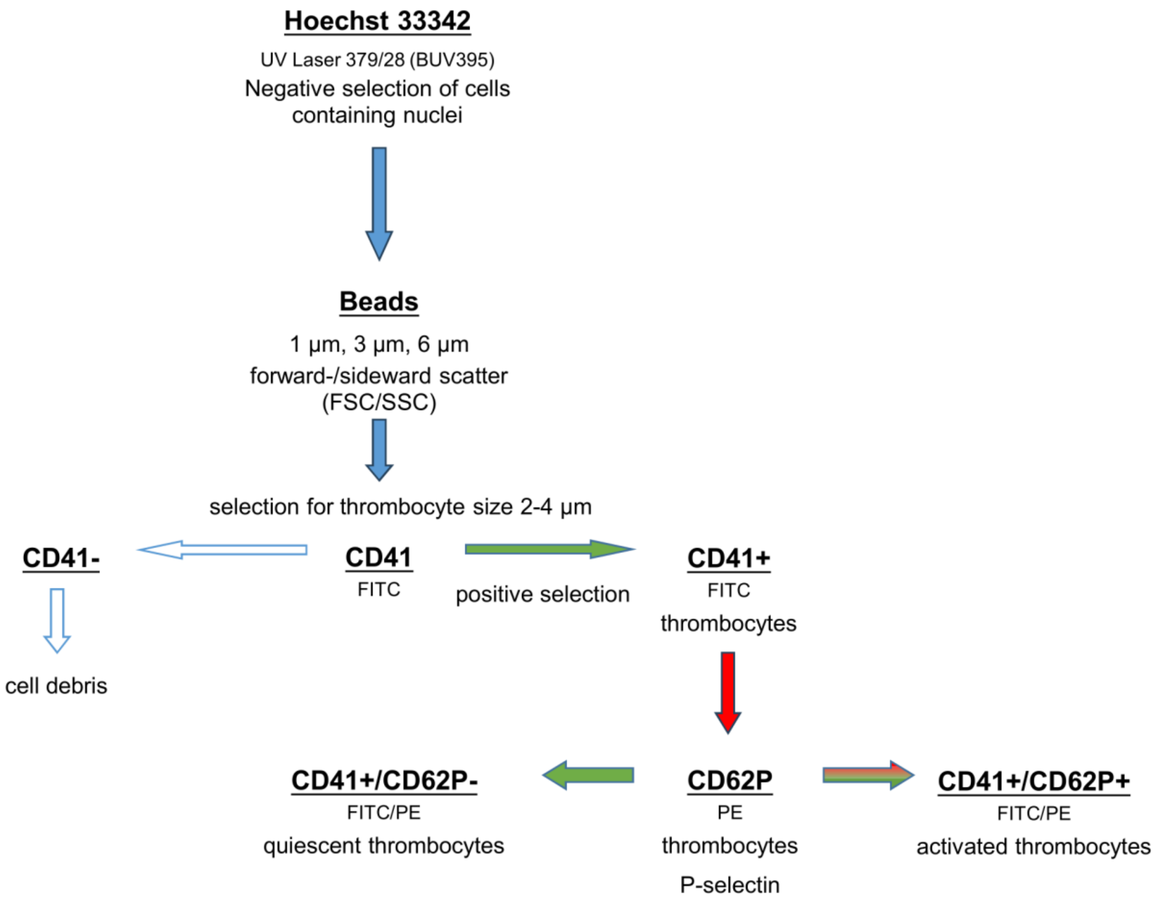

In a first step, nuclei-containing cells were excluded by nuclear staining with Hoechst 33342. Then, thromobcytes were enriched by size exclusion. Finally, total thrombocytes were selected by the expression of CD41, and activated thrombocytes by co-expression of CD62P. For further details, cf. main manuscript.

Figure S2. hBM-MSC attenuate deterioration of hepatic tissue integrity after two-thirds partial hepatectomy

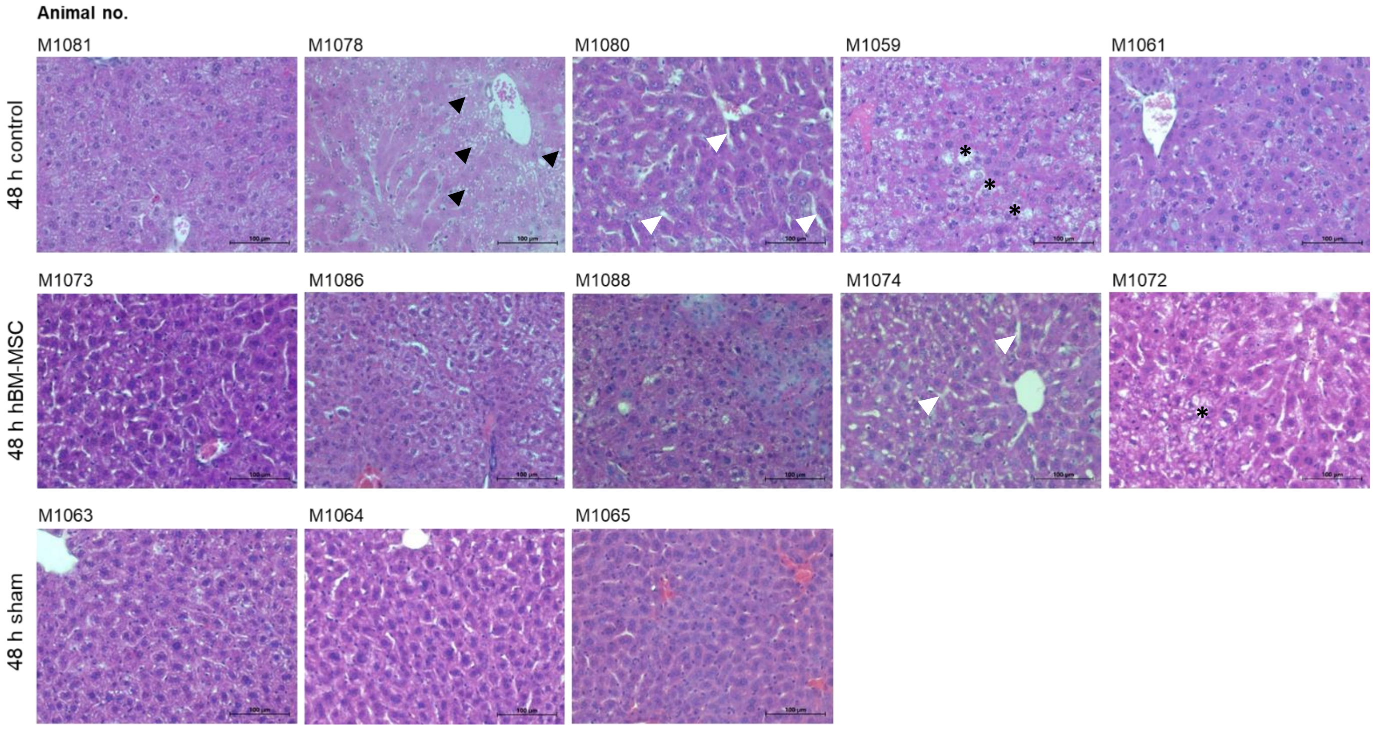

After partial hepatectomy, C57BL/6J mice were either left untreated (control) or treated with hBM-MSC. In the sham-treated group, laparatomy and closure was performed, only. Livers were collected 48 h after surgery and paraffin sections (2 µm) stained with hematoxylin-eosin. Representative pictures were taken from 3 and 5 different animals (animal identity is indicated by numbers above images) in the sham and treatment groups, resp. Surgery-induced tissue damage is observed in the control group, which is less pronounced after hBM-MSC treatment. Black arrowheads – lipid accumulation; white arrowheads – widened sinusoids; asterisks – necrotic hepatocytes.

Figure S3. Immunohistochemical detection of proliferating liver cells after two-thirds partial hepatectomy by Ki67 staining

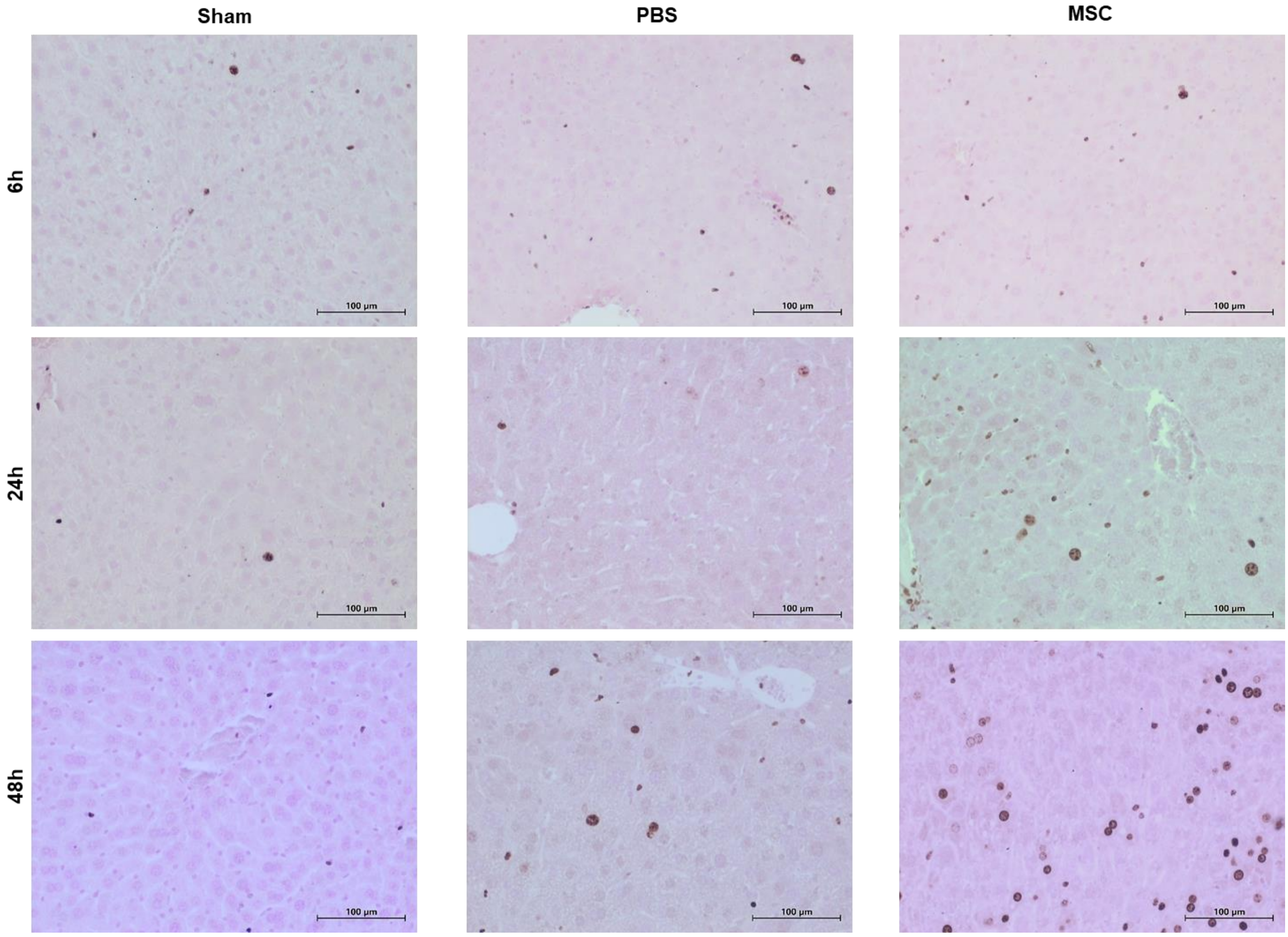

Immunohistochemical detection of Ki67 demarcating proliferating liver cells (brown staining) at the times indicated after partial hepatectomy. Pictures were used to determine the amount of proliferating cells out of total hepatic cells as shown in Figure 1B in the main manuscript. Pictures shown are representative for 3 animals in the sham and 5 animals in the control and the hBM-MSC-treated group each. 15 microscopical fields (orig. magnification – 20x) were counted and used for quantification with the ImageJ software as described in the Materials and Methods section of the main manuscript.

Figure S4. hBM-MSC treatment does not decrease blood levels of TGF-β3 after two-thirds partial hepatectomy

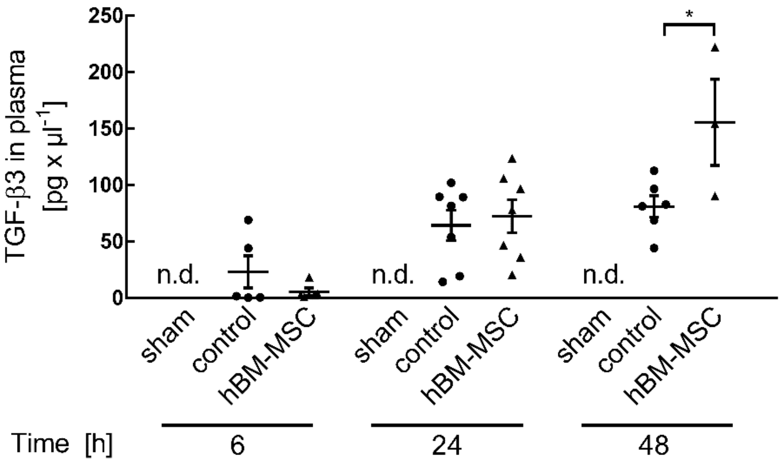

TGF-β3 was measured by ELISA in plasma supernatants after centrifugation of blood samples taken at the time points indicated. Values are means ± SEM from 3 (sham) and 3-7 animals in the control and hBM-MSC groups, respectively, as indicated by the single data points. To assess for data normality, the Shapiro-Wilk and the Kolmogorov-Smirnov tests were used. The tests confirmed normal distribution. Further statistical analysis between the different treatments was performed using the Student's t-test. Values are significantly different at the *P*-level of *≤0.05. n.d. – not determined.

Figure S5. hBM-MSC attenuate deterioration of tissue integrity after two-thirds partial hepatectomy

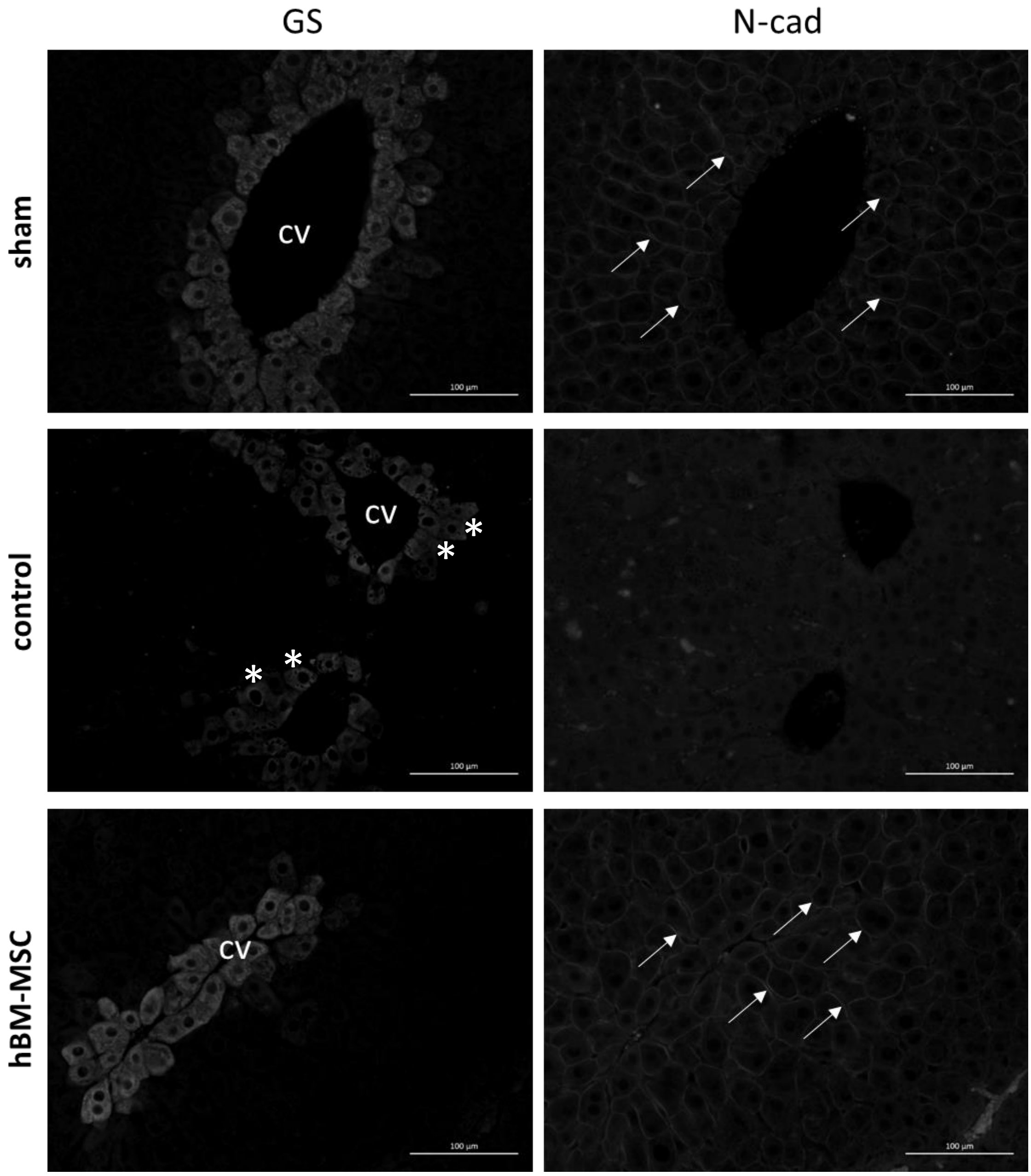

Immunofluorescent co-detection of N-cadherin (left) and glutamine synthetase (GS, right) in liver tissue sections 48 h after partial hepatectomy. While N-cadherin demarcates epithelial cell-cell contacts predominantly in hepatocytes lining the proximal branches of the central vein (cv), GS is expressed in a few cell layers of hepatocytes surrounding the central vein. N-cadherin expression indicates epithelial cell-cell contacts between hepatocytes in sham-treated animals (right upper panel; white arrows). Partial hepatectomy diminishes abundance of N-cad, which is hardly visible. Expression of GS in pericentral hepatocytes becomes blurry (left middle panel, asterisks). Treatment with hBM-MSC restores expression of GS (left lower panel) and N-cad (right lower panel) comparable to sham-treated animals. Images shown are representative for different tissue slices from 2-5 mice out of the different groups. Microscope – Zeiss Axio Observer.Z1. Original magnification – 20x. Scale bar – 100 µm. cv – central vein.

Figure S6. hBM-MSC attenuate thrombospondin-1-positive platelets in the liver after two-thirds partial hepatectomy

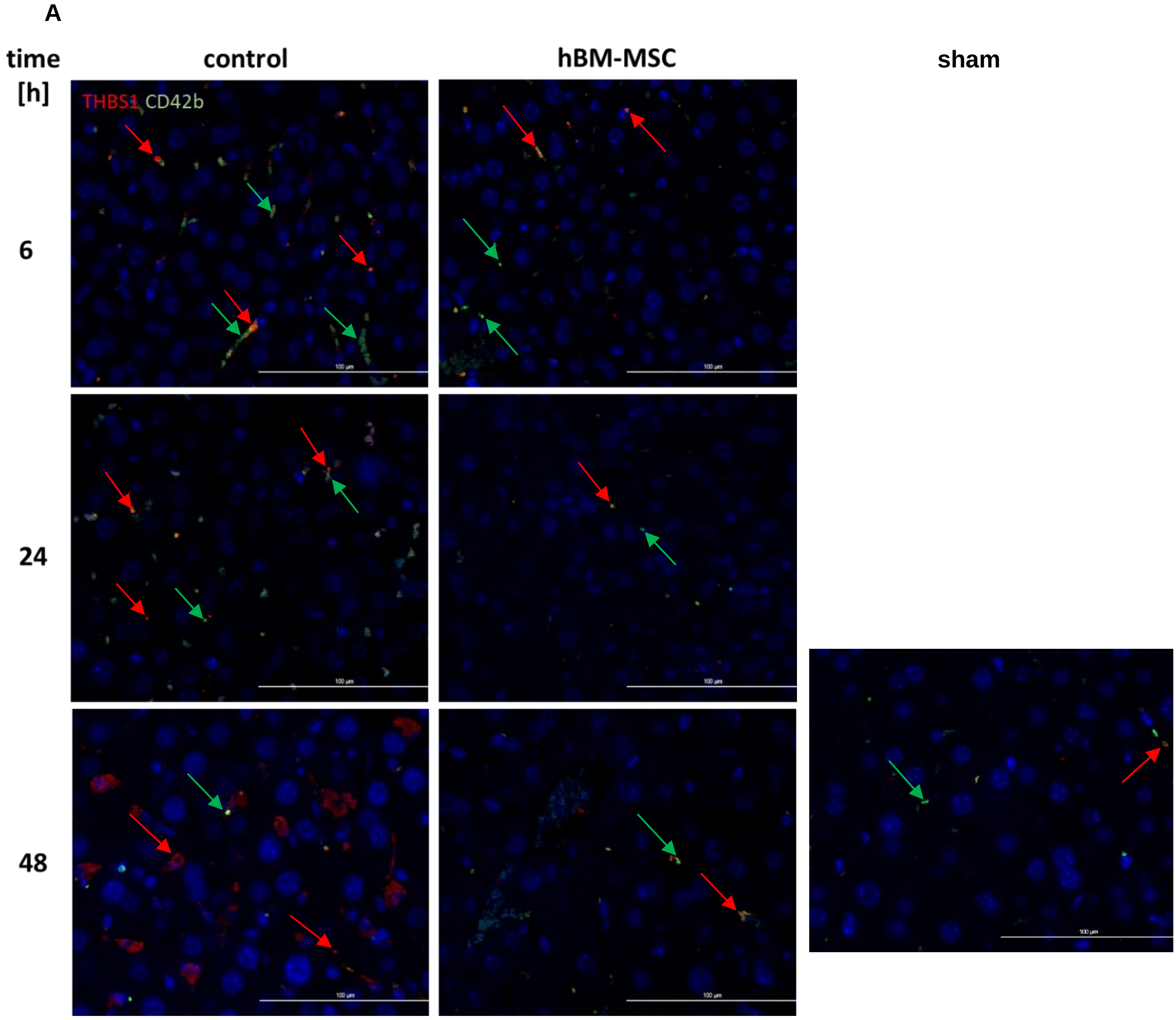

Platelets were marked with CD42b at the times indicated and detected by immunofluorescence (green fluorescence, green arrows). Red fluorescence indicates THBS1 (red arrows). Co-localization of the two markers delineates thrombocytes as potential source of THBS1. At any point in time, thrombocytes are obviously diminished by the treatment with hBM-MSC. At 48 h after partial hepatectomy, besides platelets other cells are detected by the THBS1 stain indicating a different cell source of THBS1. Images shown are representative for different tissue slices from 2-6 mice out of the different groups. 7-27 microscopic fields from the different groups were used for quantitative image analysis of data shown in Figure 4 of the main manuscript. Microscope – Zeiss Axio Observer.Z1 with ApoTome.2. Original magnification – 40x. Scale bar – 100 µm.

Figure S7. hBM-MSC attenuate the increase of macrophages in the liver after two-thirds partial hepatectomy

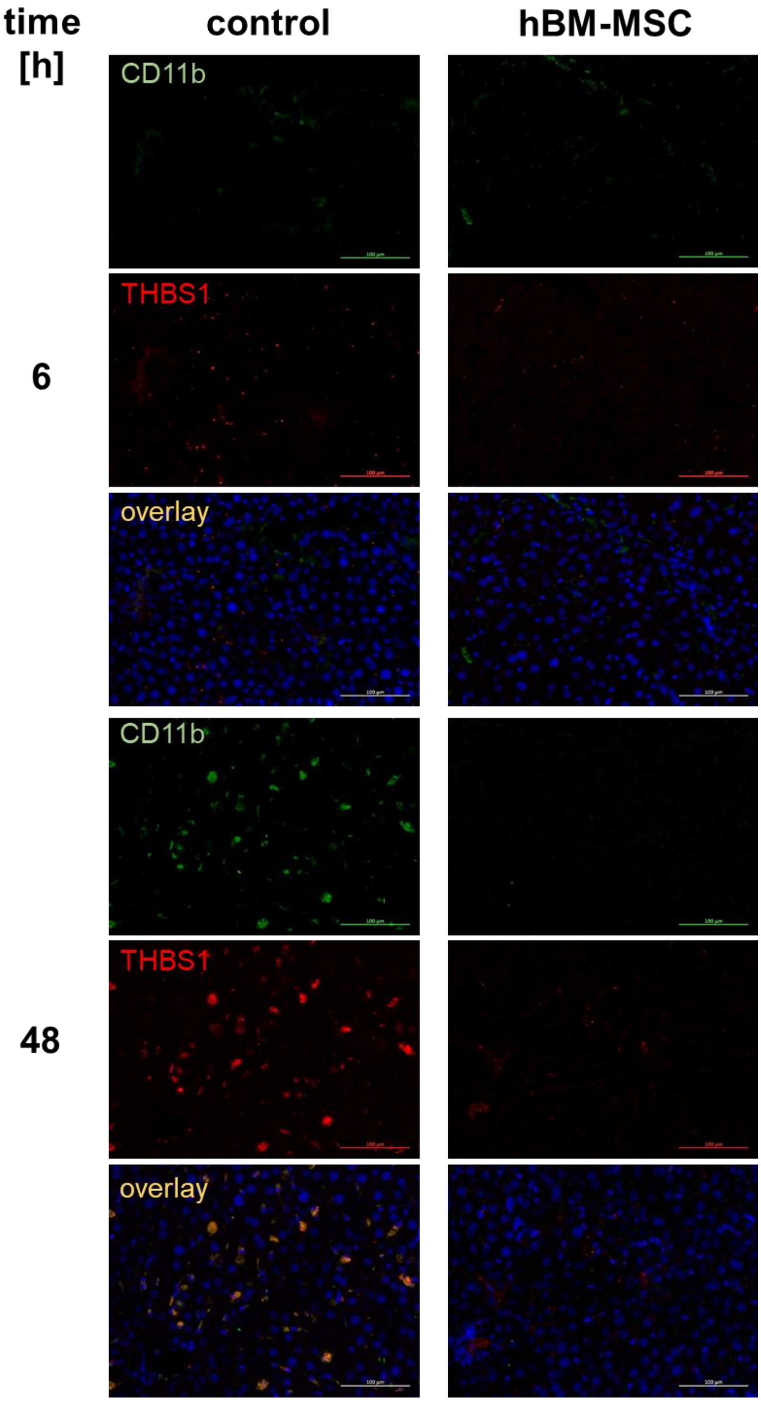

Macrophages were marked with CD11b at the times indicated and detected by immunofluorescence (green fluorescence). Red fluorescence indicates THBS1. Co-localization of the two markers delineates macrophages as potential source of THBS1. At any point in time, macrophages are obviously diminished by the treatment with hBM-MSC. Images shown are representative for different tissue slices from 3 mice out of the different groups. Microscope – Zeiss Axio Observer.Z1 with ApoTome.2. Original magnification – 20x. Scale bar – 100 μm.

Figure S8. Tissue morphology and abundance of THBS1 in livers of patients undergone the ALPPS procedure

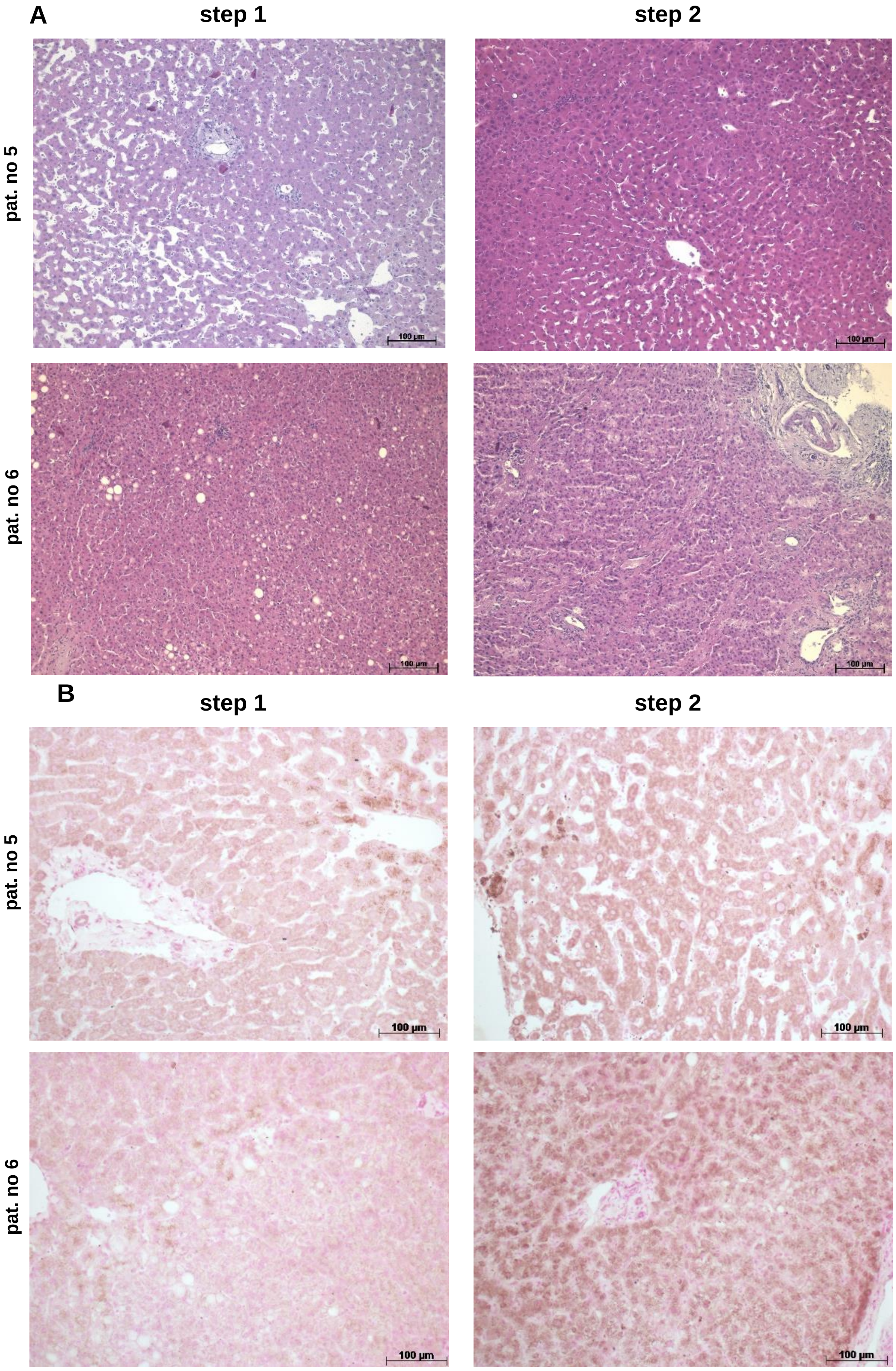

In (A), tissue morphology was depicted by HE staining and in (B), THBS1 was detected by immunohistochemistry. Tissue samples were taken at the time point of step 1 representing the situation at the starting point, and at step 2 featuring the time point of resection of the ligated liver lobe(s). At step 2,

biopsies were acquired from the future liver remnant. Microscope – Zeiss Axio Observer.Z1. Original magnification – 10x (A), 20x (B). Scale bar – 100 µm.

Figure S9. Peri-operative time course of blood levels of albumin and the activated Partial Thromboplastin Time (aPTT) in patients undergone the ALPPS procedure

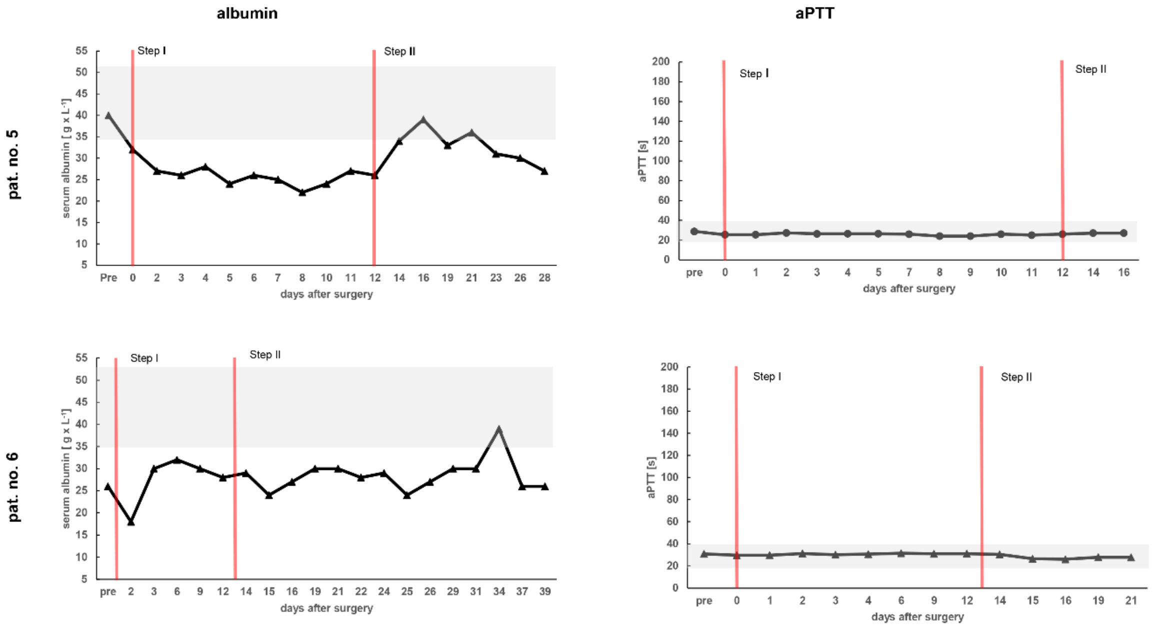

During the ALPPS procedure, albumin and the aPTT were determined in the patients´ blood samples at the times indicated. The time points of step 1 and step 2 of the ALPPS procedure are marked with red vertical lines. Grey areas demarcate the range of physiological blood values.

Supplementary Methods

Table S1. Summary of primer pairs and conditions used for semi-quantitative RT-PCR

Gene Name	Primer Sequences [5' → 3']	T_A [°C]	Product Size [bp]
B2M (β-2-microglobulin)	F: TCTACTGGGATCGAGACATGTGA<br>R: ATTGCTATTTCTTTCTGCGTGCAT	60	124
CDH1 (E-cadherin)	F: GGACGTCCATGTGTGTGACT<br>R: GATCAGAATCAGCAGGGCGA	60	134
TJP1 (ZO-1)	F: AGTTTCGGGTCCGAGGAG<br>R: CCCAGGAGCCCTGTGAA	60	137

F: forward primer; R: reverse primer; T_A: annealing temperature; bp: base pairs.

Table S2. Summary of antibodies and their dilutions used in this study

Antibody	Species	Dilution	Cat. No.	Supplier
THBS1	mouse	1:100	ab1823	Abcam, Cambridge, England
CD42b	rabbit	1:200	ab183345	Abcam, Cambridge, England
CD11b	rabbit	1:200	ab133357	Abcam, Cambridge, England
E-cad	mouse	1:200	610182	BD, Heidelberg, Germany
N-cad	mouse	1:200	610920	BD, Heidelberg, Germany
ZO-1	rabbit	1:100	40-2200	InVitrogen, Carlsbad, CA, USA
CYP2E1	rabbit	1:100	ab28146	Abcam, Cambridge, England
GS	mouse	1:1000	610518	BD, Heidelberg, Germany
GS (N-cad co-stain)	rabbit	1:500	Ab73593	Abcam, Cambridge, England
Ki67	rabbit	1:200	ab66155	Abcam, Cambridge, England
Biotin-SP-conjugated anti-rabbit	goat	1:200	111-065-003	Dianova, Hamburg, Germany
AlexaFluor 488-labeled anti-rabbit	goat	1:200	A11008	Thermo Fisher Scientific, Dreieich, Germany
Cy3-labeled anti-mouse	goat	1:200	115-165-003	Dianova, Hamburg, Germany
Biotin-labeled anti-mouse	goat	1:200	115-065-003	Dianova, Hamburg, Germany

Table S3. Summary of antibodies used for flow cytometry

Antibody	Label	Clone	Cat. No.	Supplier	Dilution
Hu/mo anti-CD62P (P-Selectin)	allophycocyanin (APC)	Psel.KO2.3	17-0626-82	Thermo Fisher Scientific, Darmstadt, Germany	1:200
Mouse IgG1 kappa Isotype Control	allophycocyanin (APC)	P3.6.2.8.1	17-4714-42	Thermo Fisher Scientific, Darmstadt, Germany	1:200
rat anti-mouse CD41	fluorescein isothiocyanate (FITC)	MWReg30	553848	BD Pharmingen, Heidelberg, Germany	1:200
Rat IgG1, κ Isotype Control	fluorescein isothiocyanate (FITC)	R3-34	553924	BD Pharmingen, Heidelberg, Germany	1:200

Table S4. Summary of serum transaminases after two-thirds partial hepatectomy.

Mouse identity	Treatment	Time [h]	AST [µkat/l]	ALT [µkat/l]	De-Ritis-q. [AST/ALT]	Mean	SEM	*P*[1]	
M1053	sham	6	4	n.d.	-				
M1054	sham	6	4	n.d.	-				
M1055	sham	6	4	n.d.	-				
						-	-		
M1025	control	6	48	44	1.09				
M1026	control	6	48	52	0.92				
M1039	control	6	48	52	0.92				
M1040	control	6	38	32	1.19				
M1046	control	6	34	28	1.21				
M1047	control	6	84	26	3.23				
M1048	control	6	54	22	2.45				
						1.57	0.34		
M1027	hBM-MSC	6	78	90	0.87				
M1032	hBM-MSC	6	68	54	1.26				
M1033	hBM-MSC	6	78	72	1.08				
M1034	hBM-MSC	6	62	24	2.58				
M1035	hBM-MSC	6	104	72	1.44				
M1050	hBM-MSC	6	32	24	1.33				
M1051	hBM-MSC	6	36	30	1.20				
						1.40	0.21	0.39	vs. 6h control
M1056	sham	24	2	n.d.	-				
M1057	sham	24	2	n.d.	-				
M1058	sham	24	30	16	1.88				
						-	-		
M1041	control	24	120	38	3.16				
M1042	control	24	176	50	3.52				
M1043	control	24	224	80	2.80				
M1044	control	24	56	26	2.15				
M1045	control	24	162	34	4.76				
M1052	control	24	78	38	2.05				
M1083	control	24	250	114	2.19				
M1084	control	24	172	76	2.26				
						2.86	0.33	0.003	vs. 6h control
M1028	hBM-MSC	24	218	106	2.06				
M1029	hBM-MSC	24	n.d.	70	-				
M1030	hBM-MSC	24	174	106	1.64				
M1031	hBM-MSC	24	50	120	0.42				
M1036	hBM-MSC	24	326	138	2.36				
M1037	hBM-MSC	24	n.d.	102	-				
M1038	hBM-MSC	24	144	58	2.48				
M1089	hBM-MSC	24	132	66	2.00				
M1090	hBM-MSC	24	200	104	1.92				
						1.84	0.26	0.02	vs. 24h control

M1063	sham	48	n.d.	n.d.	-				
M1064	sham	48	n.d.	n.d.	-				
M1065	sham	48	2	n.d.	-				
						-	-		
M1059	control	48	26	14	1.86				
M1061	control	48	214	70	3.06				
M1062	control	48	204	78	2.62				
M1078	control	48	118	32	3.69				
M1079	control	48	194	28	6.93				
M1080	control	48	38	24	1.58				
M1081	control	48	202	50	4.04				
						3.40	0.68	0.01	vs. 6h control
M1066	hBM-MSC	48	22	14	157				
M1072	hBM-MSC	48	6	6	1.00				
M1073	hBM-MSC	48	4	4	1.00				
M1074	hBM-MSC	48	30	8	3.75				
M1075	hBM-MSC	48	8	6	1.33				
M1077	hBM-MSC	48	90	30	3.00				
M1086	hBM-MSC	48	n.d.	146	-				
M1088	hBM-MSC	48	n.d.	156	-				
						1.94	0.47	0.04	vs. 48h control

[1] Statistics: Values are means ± SEM from the number of animals as indicated. To assess for data normality, the Shapiro-Wilk and the Kolmogorov-Smirnov tests were applied. The tests revealed a deviation from normal distribution and a Johnson transformation was performed. Further statistical analysis between the different treatments were performed using the Student's t-test. Values are significantly different at the *P*-level as indicated.

n.d. not detectable; - not determined.
